# A Practical Account of the Mediterranean Fever, &c. &c.

**Published:** 1816-09

**Authors:** 


*A Practical Account of the Mediterranean Fever, &c. &c.* By William Burnett, M. D. &c. &c. &c. Second Edition, greatly enlarged. London, 1816.

The first edition of this work was so extensively reviewed by us, that we can only notice some of the new matter which has been added to the present, particularly the seventh appendix, containing a long and very important answer to Dr. Pym. In this discussion, Dr. Burnett has very judiciously expressed himself in temperate language, and avoided all acrimonious and petulant remarks.

He commences by exculpating himself from all intention of giving offence to the medical officers of Gibraltar, in stating, that their treatment of the fever in 1804 had been "any thing but successful." The fact of half the population having been destroyed, and the confession of Sir James Fellowes himself, bear out Dr. Burnett's assertion.

Dr. Burnett next proceeds to examine Dr. Pym's *distinctions* between *Bulam* and bilious remittent fever, first shewing that the pulse is *res fallacissima;* and next, that, contrary to Dr. P's assertion, the Bulam fever "*not only bears, but requires venæsection for its cure.*" He refers to the account published by Mr. Humphreys, in the Edinburgh Journal, where blood-letting was eminently successful, and to which we refer our readers.

It appears also, that Mr. Lea, surgeon of the 26th regiment, "bled seventy-eight of his patients, not one of whom died."

"My general plan," says he, "while I had charge of the 26th regiment, and what I observed practised by my particular friend, Surgeon Weld, of the 67th, was bleeding and copious purging, which was attended with the happiest effect."

This was in 1814; and Dr. M'Millen, who had ample experience in the West Indies,

"*After an examination of the sick, in the town and garrison of Gibraltar, and the appearances on dissection after death, pronounced the disease to be the yellow fever of the West Indies, and not contagious.* He immediately ordered *bleeding to be employed freely at its commencement, and almost every patient recovered with whom this remedy was used.*" 354.

Mr. Martindale, Assistant Surgeon of the 67th Regiment, reports to the Medical Board, that the lancet "appeared of great benefit, *if* employed within twenty-four hours after the accession of the fever."

Surgeon Sproule, of the Royal Artillery, acknowledges that he did not bleed, but candidly confesses—

"At the same time, from the appearances on dissection, I think I have reason to regret not having had recourse to that mode of treatment."

Amongst the *post mortem* appearances were—"great turgescence of the vessels of the brain." The practice of blood-letting was confined to the 26th, 67th, and that portion of the Ordnance service under Mr. Humphreys; in these it was eminently successful.

Dr. Pym has quoted the authority of Dr. Moreno (the last Spanish writer on the disease) against blood-letting; the following passage from the same author affords no proof of Dr. Moreno being in the right.

"Semegantes hæmorrhagias (epistaxis) en il primer periodo de la enfermedad, son casi siempre saludabales pues desminuyen algun tanto la reaccion del systema que pertenecen, alivian el dolor de Cabeza, haeen se presente el pulso mas blanda é inclinan la cutis al sudor."*

One of Dr. Pym's characteristics of the Bulam is, that it "*generally* terminates before the 5th day, and is very often attended with the fatal symptom of the black vomiting." By reference to page 249 of this gentleman's book, it will be found, that out of a list of eighty-nine cases of this fever, which he says he attended in 1804, at Gibraltar, only *five* became convalescent *before* the fifth day; "so that his own statements, says Dr. B. contradict his assertions."

Mr. Donnet, of the Naval Hospital (a gentleman whom Dr. Pym eulogises) sent Dr. Burnett six prescription tickets of cases in 1814; which, on examination, give 4, 8, 9, 11, 12, 15, days before death or convalesence. P. 417.

Mr. Short, Surgeon of the 60th Regiment, in reply to the official queries of Mr. Fraser, states, that,

"Of seventeen fatal cases, one died on the 4th, one on the 5th, nine on the 6th, two on the 7th, two on the 8th, and one on the 10th day of illness."

Mr. Barker, of the 11th Regiment, observes, that the fatal cases terminated—

"One on the 3d, one on the 4th, one on the 5th, one on the 9th, and one on the 10th day."

Assistant-Surgeon Williams, who had charge of the Lazaretto, remarks, that the fatal days were the 5th, 6th, 7th, 8th, 9th, and 10th.

Dr. Chisholm, at page 142 of first edition of Essay on *Bulam*, says,

"Thus, if the patient was worse on the evening of the second

---

* Which we shall translate thus:—"These hæmorrhages (epistaxis for instance) in the first period of the disease, are almost always salutary, by diminishing the reaction of the system, alleviating the pain of the head, rendering the pulse more soft, for a time, and inclining the skin to perspiration."—Rev.

day, he would die on the third; if worse on the fourth day, he would die on the fifth; and so on, as far as the fourteenth day. Beyond that period, I have not seen an instance of the disease ending fatally, although it has been protracted, in a few instances, to the 21*st day*."

These documents are certainly extremely unfavourable to the accuracy of Dr. Pym's statements.

As to the black vomit, it has been observed in all bad cases of ardent fevers in hot climates, since the days of Hippocrates. Cleghorn notices "*black matter like the grounds of coffee*," as a bad symptom in the diseases of Minorca fifty years ago.

Don Antonio Vilaseca, published at Majorca, an Account of the fever in the Temeraire, Invincible, and transports, declaring it the *true contagious yellow fever*. We need not say that *this* fever has been proved to be not contagious.

That the occurrence of black vomit in the *Bulam* was not so frequent as Dr. Pym would have it believed, Dr. B. brings forward the following evidence. Mr. Martindale, of the 67th Regiment, says—

"Vomiting in some, was present from the commencement to the termination; but in others, it only came on with the second stage; the fluid thrown up was, in one instance, a dark brown; but in the remainder, who died, I did *not once see the black vomit*."

Surgeon Weld, in his official replies, does not once mention the black vomit.

We now come to a very important document, which it behoves Dr. Pym to take into his most serious consideration; for unless he prove it *non-authentic*, we confidently predict, that it will go far to draw a shade over his character, as a man and an author.

Mr. Gardiner, late Surgeon of Gibraltar Hospital, has transmitted to Dr. Burnett the following paper, *written by Dr. Pym himself*, on the Bulam of 1804; (since which he has had little opportunity of seeing the disease) and which, if compared with Dr. Pym's description of the disease, *in his printed work*, will be found to involve the most important contradictions; and will, we fear, give too much reason for Dr. Burnett to say, that

"The *marked* difference in the method of treatment he *now* advises, and that which he tells us he actually practised, induces me to believe, that he is more indebted to Callow's Library, than to any observation he made on the spot." 428.

*Description of the* BULAM *of* 1804, *by Dr. (then Mr.) Pym.*

"The disease generally comes on like other fevers, with a slight head-ache; chilliness and shivering; sometimes sickness at stomach; pulse from 100 to 130. These symptoms are, in a few hours, followed by violent pain in the head, *confined chiefly to the eye-balls and forehead*, back and calves of the legs; *the face becomes flushed, and the eyes have a shining and watery appearance, with a slight degree of inflammation, like those of a person half drunk.* The skin dry; the bowels in general bound; tongue foul, with a considerable degree of thirst.

"This is most commonly the first stage of the disease; but sometimes, the patient is seized in a moment with the violent head-ache, pain of the back, and the greatest debility, from being apparently in the most perfect health.

"In general, when I am called in, in the early stage of the disease, I order an emetic of antimon. tartarizat. or ipecac. that is, when the patient has sickness at stomach, or an inclination to vomit, which generally produces perspiration, and sometimes opens the bowels also; if it does not, a gentle purgative must be given. When there is no sickness at stomach, I generally order a purgative, such as salts, cremor. tartar, or calomel and jalap, with an injection to hasten its operation. When this has operated three or four times, I order small doses of James's powder, such as half a grain every two hours, to produce or encourage perspiration, which, with the assistance of cooling drinks, and occasional blisters to the forehead and temples, and nape of the neck, put an end to the disease *about the third day;* when the patient must be supported with broths, wine, and jellies, given in small quantities, and frequently repeated; at the same time keeping the bowels regular, and giving occasionally small quantities of infusion of bark.

"This is the termination of what we call a mild attack; *but, in many cases, the patient goes on thus for four, or perhaps to the fifth day, without any bad symptoms,* when his pulse suddenly sinks, his eyes become yellow; bleeding takes place from the nose, mouth, and bowels, sometimes even from the eyes; his neck and arms are covered with small dark-coloured spots; there is no secretion or excretion of urine; a vomiting of a black matter, like coffee grounds, comes on, with an uneasiness in the chest, and difficulty of breathing, which puts an end to the disease, as you may suppose, about the fifth or seventh day. In this last case, we have been very seldom successful: bark, in different forms, has been used with stimulants, such as wine, brandy and water, lavender drops, with hot fomentations to the loins and region of the bladder; also rubbing the loins with hot brandy.

"*In some cases,* the patient is seized from almost the first moment of his attack, with delirium and vomiting, which soon terminates in what is called the black vomit, and death takes place on the second or third day. In this case, blisters to the head, mustard to the feet, keeping the bowels open by glysters, and giving the saline mixture, or any cooling drink, in very small quantities, have sometimes proved successful; and when the fever goes off, the greatest care is necessary to counteract the debility, as all the convalescents are liable to faint upon the smallest exertion, even upon sitting up in bed. This is best done by the different preparations of the bark, and humouring the patient with any kind of food that is most agreeable, in small quantities at a time, and frequently repeated.

" *The most characteristic symptom of the disease is the peculiar pain in the forehead and eye-balls, with the drunken appearance of the eye.*

" In the after stages, it is known, when too late, by the black vomiting, suppression of urine, hiccough, yellow skin, with small purple or blackish spots, chiefly on the neck and arms; any one of which symptoms, excepting the yellow and spotted skin, is generally looked upon as fatal. *The black vomiting has been cured, in some instances, by only wetting the patient's mouth with drink, and giving repeated injections of cold chicken broth, without salt*; and, in one case, the patient was cured by drinking capillaire and water only. The want of secretion of urine, is, I believe, always fatal. The hiccough has been got the better of by ten drops of laudanum and twenty of æther, given every two hours, for eight or ten hours."

To have a complete view of the discrepancies of *treatment* (to which we shall confine ourselves) between the above account and that in his work, from page 228 to 235, the reader must compare the two documents; but we shall point out sufficient to excite some most unpleasant sensations in the mind of every inquirer after truth, and every lover of candour!

In Dr. Pym's *book*, (p. 228) he recommends a *brisk* purgative, and sometimes a *gentle* emetic, as a glass of tepid water or weak chamomile tea; but in his *practice*, the scene is reversed!—it is then, " I order an emetic of *antim. tart.* or ipecac.——and a " *gentle purgative*.

In the *book*, he advises the Physicians of Carthagena to give calomel purgatives in much larger doses than they were accustomed to administer, and to sponge the body frequently with cold water; but in *practice*, no mention is made of the cold affusion!

In the *book*, not a word is said of *James's powder;* but in *practice*, *half a grain every two hours* appears the universal rule.

In the *book*, " I must confess," says Dr. Pym, " that I have very little opinion of it (bark) as a medicine." In *practice*, " bark, in different forms, has been used with stimulants, such as wine, brandy and water, &c. &c."

As for Dr. Pym's denunciations against venæsection in this fever, it is now well known, that he and his partisans never gave it a *fair trial;* and that others have found it eminently useful. Vide *Edinburgh Medical and Surgical Journal, &c.*

Will any man say, at *this æra,* that where we have "*violent pain in the head, confined chiefly to the eye balls and forehead, flushed face, eyes shining and watery, with a slight degree of inflammation, like those of a person half drunk,*" we are not to bleed, whatever may be the name of the disease? Yet, such are the symptoms from Dr. Pym's own description in 1804!

At the conclusion of the epidemic fever of 1814, in Gibraltar, Mr. Frazer, surgeon-major of the garrison, proposed a series of questions to the different medical officers; and the following is the result relative to the origin and subsequent propagation of the disease, extracted from their official replies: viz.

| | | |
|---|---|---|
| Surgeon Weld, 67th Reg. | Domestic Origin | Non-contagious. |
| Assist.-surgeon Martindale | ditto | ditto |
| Surgeon Lea, 26th Reg. | ditto | ditto |
| ——— Short 60th ditto | ditto | ditto |
| ——— Playfair, Dillon's Reg. | ditto | ditto |
| Assist.-surgeon, Dillon's ditto | ditto | ditto |
| Hospital Assistant Thompson | ditto | ditto |
| Surgeon Humphreys, Royal Artil. | ditto | ditto |
| Assist.-surgeon Williams | Neutral | Neutral |
| Hospital Assistant | ditto | ditto |
| Mr. Donnet, Naval Hospital | Domestic Origin | Contagious in limited degree |
| Mr. Amiel* | ditto | ditto |
| Assistant-surgeon Brady | ditto | Contagious |
| ——————— Foote | ditto | ditto |
| Surgeon Sproule, Artillery | Neutral | ditto |
| ——— Barker, 11th Reg. | Imported | ditto |
| Assistant-surgeon Considini | ditto | ditto |

From this it appears, that the balance is infinitely against the *Bulam* theory.

Dr. Pym states in his work, that in the year 1810, the garrison of Gibraltar continued healthy till *the 20th of October,* when, in consequence, as he supposes, of a breach of quarantine laws, a Minorcan family was attacked with the *Bulam.* He states, that the *first* taken ill was Jacinto Rey, a carpenter in the dock-yard, whom he suspected of having communication with the infected

* Vide this Gentleman's excellent paper in our Journal.

transports in the bay. A Spanish priest is also stated to have taken the fever by visiting this carpenter. From this source Dr. Pym traces the contagion of that season.

But Dr. Burnett brings forward evidence to prove, 1st. That Jacinto Rey, the Minorcan, was *not* the *first* person attacked. 2d. That no communication with the transports from Carthagena took place. 3d. That Dr. Pym knew of the yellow fever being on the Rock previously to the 20th of October. 4th. That no Spanish priest had ever attended Jacinto Rey. 5th. That all the men taken ill in the barrack-room of the 7th veteran battalion were *within* the walls of the town, in a very populous part. 6th. That the fever existed in the hospital of the said veteran battalion, from the 11th till the 30th of October, without evincing any contagious character.

To substantiate these positions, Dr. Burnett proceeds thus: 1st. Instead of *Jacinto Rey*, Mr. Donnet asserts, that *Mrs. Vaughan*, wife of the commissioner's clerk, was first taken ill on the *5th of October*, and died on the 13th, "with all the worst symptoms of the genuine yellow fever." Mr. Donnet also proves the 3d position, by declaring that *he reported the case to Dr. Pym himself*, though the Doctor in his work makes Jacinto Rey, at a subsequent period, the original source of the fever on shore!—Two days previous to the occurrence of this case, Mr. Amiel met with one nearly similar, and which he asserts to be the same kind of fever as that under which Jacinto afterwards laboured.

"Jacinto Rey (says Mr. Amiel) was removed to the neutral ground in a convalescent state; and my having attended him very carefully *from the first attack*, enables me to declare, that his case was similar to that of Mrs. M'Lean, whom I had visited in the neighbourhood *from the 3d to the 8th of October.*"

To prove the 2d and 4th positions, Dr. Burnett brings forward the affidavit of Jacinto Rey himself, made before Judge Advocate Larpent; when he solemnly swears—

"That previous to his being attacked (on or about the 20th of October) he had no communication, directly or indirectly, with the transports then lying in the bay of Gibraltar, and lately arrived from Carthagena. And this deponent further maketh oath, and saith, that he was not visited or attended by any Spanish priest during his illness while he continued in the garrison."

From Mr. Kidstone's returns of attacks and deaths in the 7th veteran battalion (Appendix, No. 1), it appears, that Darby Ferguson was seized with the *Bulam* on the 19th of October, *within the walls of the town*, in a very populous part, the cooperage range, as were all the others in the same corps. These were sent to the regimental hospital in the south, and continued there till the 29th of the month, "*without any precaution or separation being observed, yet no one was infected.*" On the 29th, the regimental and hospital establishment were removed to the neutral ground, after which (30th) only one case occurred, when the fever ceased!

Dr. B. justly observes, that,

"Had it been a contagious disease, it is *impossible* that it could have existed in a barrack situated in a part of the town so well inhabited, or in the hospital of the regiment, for *nineteen days*, where no precaution was used, without, in the one case, attacking some of the other soldiers in the barrack, or the surrounding inhabitants; or in the other, infecting some of the hospital attendants or sick." 453.

With respect to two cases of *black vomiting* in the veteran battalion, Dr. B. positively avers, that the late Mr. M'Affeer, surgeon of the regiment, repeatedly told him no such thing occurred. Mr. Gardiner heard the same declaration of the deceased gentleman. This is another accusation, which it is incumbent on Dr. Pym to wipe off, as Mr. Gardiner may be appealed to.

"It is evident (says Dr. B.) that the disease in question did not radiate from any particular place, but arose simultaneously in several persons living at a distance, and perfectly unconnected with each other. As instances, I may mention Darby Ferguson, who was taken ill on the 19th of October, *within the walls of the town*; and Jacinto Rey, who was attacked on the 20th in the neighbourhood of Scud Hill. The disease certainly appeared first among the men newly arrived from England, then on board the San Juan in the Mole. The case of Mrs. M'Lean, mentioned by Mr. Amiel, seems to have been the next; then Mrs. Vaughan's; and subsequently it became more general, attacking ten soldiers of the 7th veterans *quartered in the town*, and *not* in the 'infected district,' as Mr. Pym will have it; so that, instead of '*only two* being taken ill within the walls of the town,' there were no less than twelve!" 464.

Dr. B. justly observes here, that the Contagionists lay much stress on the circumstance of the disease running through whole families, not considering that all the members of a family would probably be exposed to the same remote cause. But, in fact, the fever does not always run in this way.

"I have known (says Mr. Martindale) several instances, where only one in a family, or even in a whole house, was taken ill; of ten officers in the 67th regiment, who had fever, not one of their servants took it in consequence." 466.

Dr. Burnett allows, that those who have passed through the concentrated forms of yellow fever, are less liable to secondary attacks than others who have not, especially while they continue in the same atmosphere, or in a southern climate—

"—but the following list of nearly fifty authenticated instances of second attacks in the fever of Gibraltar, most of which occurred in the last epidemic, place it beyond doubt that this is not uniformly the case."

From official replies it appears, that Mr. Glasse saw 2, Mr Donnet 1, Mr. Amiel 2, Surgeon Short 7, Surgeon Lea 2, Assistant-Surgeon Brady 10, Surgeon Sproule 2, Surgeon Barker 2, Assistant-Surgeon Williams 5, Mr. Muir's Case 1. In the 26th regiment, quartered at Gibraltar in 1814, the names and particulars of 15 cases are given.

We give these instances of second attacks on the authority of Dr. Burnett and the gentlemen mentioned; but we do not vouch for their correctness: indeed, we strongly suspect, that many of the cases brought forward, either had not the concentrated or Bulam fever in the first instance, or, if they had, that the second attack was not of the concentrated form. Dr. Pym, however, will investigate this point.

Mr. Glasse's testimony runs thus, p. 333:

"Many persons assert, that they have had the disease twice; and the case of Mr. Callaghan is the most convincing, as he was attended by Dr. Nooth and Mr. Burd in 1804, and by myself in 1813, and in both instances nearly lost his life."

Mr. Whitmarsh (now assistant-surgeon at Haslar) says,

"In a case which came under the care of Mr. Donnet, the man recovered, and was discharged; about a fortnight afterwards he had an attack similar to the first."

These cases, to bring fair evidence, however, should be detailed, as the *symptoms* are the criteria.

Mr. Muir, a merchant, says Dr. B. experienced a most violent attack of yellow fever, attended with all the worst symptoms of that disease, in Surinam, in the year 1811 or 12; and thinking himself safe at Gibraltar in 1813, he was attacked, and died on the 4th day, with yellow skin and black vomit.

Our readers know, that Mr. Amiel mentions two cases under his own care, who undoubtedly had second attacks. Sir James M'Grigor, in his Sketches, states, that in the

Betsey transport, a great many were twice attacked with yellow fever.

At page 258 of Dr. Pym's work, is introduced a letter from Mr. Gardiner to the Transport Board, wherein the fever of 1810 is characterized as "a disease of a very *suspicious* type, in point of malignity of contagion." The sentence itself is rather of a "*suspicious type*," and savours somewhat of Delphic ambiguity; but Dr. Burnett accuses Dr. Pym in this instance of want of candour, in leaving out that part of Mr. Gardiner's letter, in which he (Mr. G.) declares the disease to have been of *local origin, and not imported.* But in truth, it is so rare to see party writers quote any thing against themselves, *unless they have materials at hand to damn the quotation,* that we have long since given over the hope of such candid conduct: indeed, the anti-contagionists are not behind-hand in this respect, as we could abundantly shew.

But Dr. Burnett's accusation against Dr. Pym, of *garbling* Mr. Donnet's testimony, is not of so venial a nature as the foregoing trespass; and this is another stigma, which it is highly incumbent on Dr. Pym to immediately clear off, if he can. To the official question respecting second attacks of the "*Bulam,*" Mr. Donnet answers,

"I am of opinion, that those who have had the epidemic once, are not liable to be attacked a second time; *but the case I subjoin is, I think, an exception.*"

Here follows the case, &c.

Dr. Pym here mistook a *semicolon* for a *full stop,* and left out that part of the sentence marked in italics, with the case, as *unnecessary* for his purpose. To the question, whether Mr. Donnet observed the disease assume a distinct intermittent form in any case? he answers,

"I have not seen any case of the epidemic assume an intermittent form in this place; *but when I was in the West Indies, I observed it, especially after we had departed from thence, and approached a northern latitude.*"

From this part of the sentence it is evident, that Mr. Donnet considered this fever of the West Indies, which terminated in intermittent, to be the same as the epidemic of Gibraltar; but for what reason we cannot tell, Dr. Pym has here again changed the semicolon into the period, and made a *full stop* at the commencement of the italic part of the sentence.

As our concluding limits warn us to close our observations, we shall only advert to the subject of *relapses*, and leave to Dr. Pym the task of vindicating himself from the above-mentioned charges.

Our readers will recollect what Mr. Amiel says—

"I have *frequently* observed *relapses* in the epidemic fever, after all the morbid symptoms had been overcome, debility excepted."

Surgeon Weld states, that four relapses took place in the 67th regiment in 1814. Mr. Martindale, his assistant, more particularly mentions them. Surgeon Lea, 26th regiment, says,

"I can unquestionably support, by cases and undeniable authority, instances of second attack and relapse under my observation."

While reviewing Sir James Fellowes's Reports, we there alluded to the statement of Aregula, that "relapses were *very* frequent and fatal."

This second edition of Dr. Burnett's work contains a great many original and interesting documents, which, with the mass of valuable facts in the first edition, will render it a standard publication for future generations. When war shall once more call forth our fleets and armies to the scene of this fever, our younger brethren in the profession will reap the advantage of Dr. Burnett's labours; and we are convinced his energetic practice will rescue thousands from untimely death, when he himself shall have long ceased to feel an interest in sublunary affairs. Many important points relative to the nature and origin of this fever yet remain to be settled; and, much as we are averse to professional controversies, the interests of science and humanity demand a fair hearing on every side of the question. We ourselves adhere to the opinion which we have so often stated in this Journal, though it does not exactly quadrate with either the contagionists or non-contagionists. We do *not* believe the fever to be *sui generis,* or of foreign origin; and we *do* believe, that it is occasionally, or even frequently contagious, especially where accumulation and want of ventilation exist. In respect to the treatment, we believe that, upon the whole, depletion, in one shape or other, will ultimately be acknowledged the principal remedy.

As we have thought it our duty to lay before the public Dr. Burnett's reply to Dr. Pym, and especially as he has set an example of calm and dignified discussion, we are bound, as long as moderation be observed, to give Dr. Pym, whose abilities we highly respect, a fair opportunity of vindicating his opinions, and of obviating the unfavourable impression which some of his antagonist's charges must assuredly make on the public mind.

